# Blockade of Toll-like receptor 2 prevents spontaneous cytokine release from rheumatoid arthritis *ex vivo *synovial explant cultures

**DOI:** 10.1186/ar3261

**Published:** 2011-02-23

**Authors:** Sinéad Nic An Ultaigh, Tajvur P Saber, Jennifer McCormick, Mary Connolly, Jerome Dellacasagrande, Brian Keogh, William McCormack, Mary Reilly, Luke A O'Neill, Peter McGuirk, Ursula Fearon, Douglas J Veale

**Affiliations:** 1 Department of Rheumatology, St Vincent's University Hospital, Dublin Academic Medical Centre, Elm Park, Dublin 4, Ireland; 2 Opsona Therapeutics Ltd, The Trinity Centre for Health Sciences, Institute of Molecular Medicine, St James' Hospital, Dublin 8, Ireland

## Abstract

**Introduction:**

The aim of this study was to examine the effect of blocking Toll-like receptor 2 (TLR2) in rheumatoid arthritis (RA) synovial cells.

**Methods:**

RA synovial tissue biopsies, obtained under direct visualization at arthroscopy, were established as synovial explant cultures *ex vivo *or snap frozen for immunohistology. Mononuclear cell cultures were isolated from peripheral blood and synovial fluid of RA patients. Cultures were incubated with the TLR1/2 ligand, Pam3CSK4 (200 ng, 1 and 10 μg/ml), an anti-TLR2 antibody (OPN301, 1 μg/ml) or an immunoglobulin G (IgG) (1 μg/ml) matched control. The comparative effect of OPN301 and adalimumab (anti-tumour necrosis factor alpha) on spontaneous release of proinflammatory cytokines from RA synovial explants was determined using quantitative cytokine MSD multiplex assays or ELISA. OPN301 penetration into RA synovial tissue explants cultures was assessed by immunohistology.

**Results:**

Pam3CSK4 significantly upregulated interleukin (IL)-6 and IL-8 in RA peripheral blood mononuclear cells (PBMCs), RA synovial fluid mononuclear cells (SFMCs) and RA synovial explant cultures (*P *< 0.05). OPN301 significantly decreased Pam3CSK4-induced cytokine production of tumour necrosis factor alpha (TNF-α), IL-1β, IL-6, interferon (IFN)-γ and IL-8 compared to IgG control in RA PBMCs and SFMCs cultures (all *P *< 0.05). OPN301 penetration of RA synovial tissue cultures was detected in the lining layer and perivascular regions. OPN301 significantly decreased spontaneous cytokine production of TNF-α, IL-1β, IFN-γ and IL-8 from RA synovial tissue explant cultures (all *P *< 0.05). Importantly, the inhibitory effect of OPN on spontaneous cytokine secretion was comparable to inhibition by anti-TNFα monoclonal antibody adalimumab.

**Conclusions:**

These findings further support targeting TLR2 as a potential therapeutic agent for the treatment of RA.

## Introduction

Rheumatoid arthritis (RA) is a chronic inflammatory disease characterized by synovial inflammation and destruction of cartilage and bone. This process depends on cytokines and growth factors to stimulate cell survival, proliferation and extracellular matrix (ECM) degradation [1]. Activated lymphocytes play a critical role in the initiation and perpetuation of synovial inflammation. Pro-inflammatory cytokines, such as TNF-α and IL-1β, are key mediators of these processes; however, it remains unclear which mechanisms are involved in the initiation and regulation of cytokine production and other tissue-destructive mediators.

There is mounting evidence for the involvement of Toll-like receptors (TLRs) in RA [2,3]. Increased expression of TLR2 and TLR4 has been demonstrated in synovial cells and tissue [4-6]. TLR2 expression in RA synovial tissue has been demonstrated at sites of attachment and invasion into cartilage and bone [4], on CD16+ monocytes and synovial macrophages [5]. TLR2 mRNA is upregulated in RA synovial fibroblasts (FLS) by TNFα and IL-1β [4]. Overexpression of dominant negative forms of the essential TLR2/4 adapter molecules MyD88 and Mal/TIRAP inhibits cytokine production and matrix metalloproteinases in RA synovial cells [6]. Furthermore, several animal models use bacterial wall components and peptidoglycans (PG), known to activate TLR2, to induce experimental arthritis [7,8].

Targeted biologic therapies, including TNF blocking drugs, have had an important effect on the therapeutic outcome of inflammatory arthritis [9]; however, a significant proportion of patients do not respond or have a sub-optimal response highlighting the need for new therapeutic targets. TLR expression on RA cells and their ability to induce pro-inflammatory cytokines, suggest TLRs may play an integral role in the pathogenesis of RA, as such TLRs represent a rational target for therapeutic intervention [3].

In the present study, using whole tissue synovial explant cultures *ex vivo *(which closely reflect the *in vivo *environment) and RA mononuclear cells, we demonstrate that Pam3CSK4, a TLR1/2 agonist, significantly increases release of key cytokines, an effect that is blocked by an anti-TLR2 antibody, OPN301. In RA synovial explants, we demonstrate that OPN301 penetrates the synovial tissue, localizing to the lining layer and perivascular region and significantly suppresses spontaneous release of pro-inflammatory cytokines. This effect was comparable to that of Adalimumab, a well established TNF blocking therapy. Inhibition of spontaneous pro-inflammatory cytokine production by OPN301 from RA synovial explants in the absence of a specific TLR2 agonist suggests expression of endogenous TLR ligands in RA synovial tissue. These data demonstrate that TLR2 promotes pro-inflammatory and destructive processes in RA and further support the rationale of using a TLR2 therapeutic blockade.

## Materials and methods

### Patients and RA synovial tissue

Patients with RA, classified according to the American College of Rheumatology criteria [10], were recruited from rheumatology outpatient clinics at St. Vincent's University Hospital (SVUH). All patients had an actively inflamed knee joint, despite current or previous therapy. All research was carried out in accordance with the Declaration of Helsinki, following approval by the SVUH ethics committee. All patients gave written informed consent. RA synovial tissue (ST) was obtained at the time of arthroscopy under direct visualization. Blood samples and synovial fluid were collected from patients at arthroscopy or clinics.

### Whole RA synovial tissue explant culture

To investigate the effect of OPN301 (a novel mouse IgG1 monoclonal anti-TLR2 antibody, Opsona Therapeutics, (Dublin, Ireland)), on cytokine production in the arthritic joint, an *ex vivo *RA synovial explant model was established. This system maintains the synovial architecture and cell-cell contact and spontaneously releases pro-inflammatory mediators [11,12]. OPN301 is an Opsona Therapeutics internal designation for the anti-mouse/human TLR2 monoclonal antibody known as T2.5. OPN301 is a mouse IgG1 antibody that selectively inhibits TLR2 signaling [13]. IC50s for cytokines measured ranged from 10 to 30 ng/ml for OPN301 and 1 to 10 ng/ml for Adalimumab (Humira, Abbott Laboratories, Illinois, USA). OPN301 cross reacts with mouse TLR2 but has been shown to be ineffective at inhibiting TLR4 or IL-1 receptor signaling in HEK293 or RAW264.7 cells [14]. OPN301 is capable of inhibiting responses using ligands specific for TLR1/2 (Pam3CSK4) and TLR2/6 responses (FSL1/HKLM) in human and murine cell lines (THP1 CD14 X blue, U937, J774), and also in human, murine and monkey blood (Opsona, unpublished observations).

For Pam3CSK4 stimulation experiments, two biopsies were obtained from each patient (*n *= 11 for IL-6 and *n *= 8 for IL-8) and each biopsy was sectioned into four separate pieces and cultured in 96-well plates in serum-free RPMI 1640 for 24 hr and then stimulated with Pam3CSK4 (200 ng, 1 μg/ml and 10 μg/ml) for a further 24 hr. For the inhibition experiments, three biopsies were obtained from each patient (*n *= 13), and then each biopsy was sectioned into four separate pieces and cultured in 96-well plates in full RPMI 1640 medium (10% Foetal Calf Serum, 20 mM of 1 mmole/litre HEPES, streptomycin (100 units/ml), penicillin (100 units/ml) and Fungizone (0.25/μg/ml)) in the presence of OPN301 (1 μg/ml), Adalimumab (1 μg/ml) or IgG isotype control (Mouse IgG1 isotype control, Opsona Therapeutics; 1 μg/ml) for 72 hr at 37°C in air with 5% CO_2. _Following incubation, supernatants were harvested and frozen at -80°C until further use, and the wet weight of each biopsy section was obtained. Cytokine production was corrected for by wet weight of the biopsy. Biopsies were then snap frozen in optimal cutting temperature (OCT) compound and stored at -80°C for histological analysis. To assess cell viability in RA synovial explant cultures following culture, the acetomethoxy derivate of calcein (calcein AM) was used as a marker of viability. It transports through the cellular membrane of live cells, where intracellular esterases remove the acetomethoxy group resulting in a strong green fluorescence. Following culture, explants were incubated in PBS/Calcein AM (1:1,000 dilution) in the dark for 15 minutes. Biopsies were washed in PBS and examined immediately under a fluorescent microscope. Synovial cells emitted a strong green fluorescence indicative of live cells.

### Immunohistochemistry

Synovial biopsies obtained at arthroscopy and RA ST obtained after explant cultures were snap frozen in OCT and stored at -80°C. 7 μm OCT sections were cut with a cryostat, placed on glass slides coated with 2% 3-amino-propyl-triethoxy-silane (Sigma-Aldrich Ireland, Ltd, Dublin, Ireland) and dried overnight at room temperature. Tissue sections were allowed to reach room temperature, fixed in acetone for 10 minutes and air-dried. Non-specific binding and endogenous peroxidase activity was blocked using 10% casein and 0.3% H_2_O_2_, respectively. A routine three-stage immunoperoxidase labelling technique incorporating avidin-biotin-immunoperoxidase complex (DAKO, Glostrup, Denmark) was used. For RA biopsies obtained directly from arthroscopy, the sections were incubated with OPN301 at room temperature for one hour. Sections were also incubated with an irrelevant isotype matched mouse monoclonal antibody (mAb) as a negative control. For the RA synovial explant sections that were cultured with OPN301 or matched IgG control antibody, the primary antibody step was omitted in order to examine if tissue penetration of OPN301 mAb could be detected. All sections were incubated with mouse secondary antibody/HRP for 30 minutes, washed in PBS and colour-developed in a solution containing diaminobenzadine-tetrahydrochloride (Sigma, St Louis, MO, USA), 0.5% H_2_O_2 _in PBS buffer (pH 7.6). Slides were counterstained with haematoxylin and mounted.

### Isolation and culture of peripheral blood (PBMC) and synovial fluid mononuclear cells (SFMC)

Blood and synovial fluid were obtained from patients undergoing arthroscopy or at clinics, and drawn into heparin containing tubes. PBMCs/SFMCs were isolated by Ficoll-Metrizoate density gradient centrifugation (Lymphoprep, Nycomed, Marlow Buckinghamshire, UK). Cells were seeded in 48-well plates, at a cell density of 200,000 to 400,000 cells/ml in full RPMI 1640 medium containing Pam3CSK4 (200 ng/ml, 1 μg/ml or 10 μg/ml) in the presence or absence of OPN301 or IgG isotype control for 6 hr. To assess viability of cells after stimulation with Pam3CSK4 and/or OPN301, PBMCs were stimulated with Pam3CSK4 1 μg and/or OPN301 1 μg for 24 hrs. PBMC cell suspension was diluted 1:20 in Ethidium Bromide Acridine Orange solution (EBAO). Viable and non-viable cells were counted using a dual-chamber hemocytometer and a UV-light microscope. No difference for cell viability was observed between control and Pam3CSK or OPN treated cells.

### Cytokine quantification

IL-6, IL-8, IFN-γ, IL-1β, and TNF-α, levels were quantified by Multiplex Tissue Culture kit (Meso Scale Discovery (MSD), Maryland, USA) or by ELISA (R&D Systems, Oxfordshire, UK) according to the manufacturer's instructions. Absorbance was measured at 450 nm in a microtiter plate spectrophometer (Dynatech MR4000, Alexandria, VA, USA) or using MSD Sector Imager 2400.

### Statistical analysis

SPSS15 system for Windows (SPSS Inc, Chicago, Illinois, USA) was used for statistical analysis. Non-parametric Wilcoxon Signed Rank test for related samples and non-parametric Friedman analysis of variance for comparison of three or more groups were performed. *P *< 0.05 was determined as statistically significant. Results are expressed as mean ± SEM unless otherwise stated.

## Results

### Patient characteristics

Clinical characteristics of the study patients who underwent arthroscopy are shown in Table 1. All patients had clinically active disease as estimated by high 28-joint count Disease Activity Score (DAS28) (4.1 ± 0.9; mean ± SD) and had a swollen, inflamed knee joint. All patients except one were biologically naive. Eight patients were receiving disease modifying antirheumatic drugs (DMARDs) - either Methotrexate or Plaquenil. In addition, three were receiving oral steroids (prednisilone ≤10 mg) and four patients were receiving NonSteroidal Anti-inflammatory Drugs (NSAIDs) only. One patient had previously received Methotrexate/Adalimumab had stopped treatment due to infection. There was no relationship between response to OPN301 and treatment.

**Table 1:** Characteristics of RA patients (*n *= 13)

Age, mean (range) years	58.07 (29 to 83)
Disease duration, mean (range) years	6.1 (0.6 to 14)
Rheumatoid factor - positive	8
- negative	5
No. of patients on DMARDs	8
No. of patients on NSAIDS	4
Physician's global assessment, 0 to 100 mm VAS	26.4 ± 25.8
ESR, mm/hr	24.8 ± 9.5
CRP, mg/dl	20.9 ± 18.7
DAS28, units	4.1 ± 0.9

### Effect of Pam3CSK4 on cytokine production

**Figure 1:**
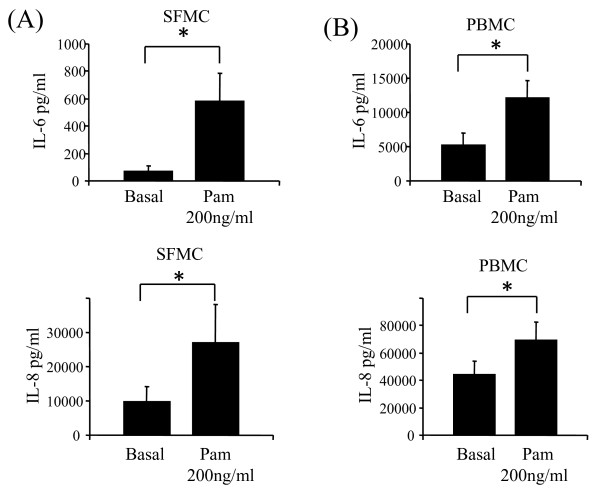
**Pam3CSK4 induced IL-6 and IL-8 in mononuclear cells from RA patients**. SFMCs (**A**; *n *= 6) and PBMCs (**B**; *n *= 11) were stimulated with TLR2 agonist Pam3CSK4 at 200 ng/ml. Levels of IL-6 and IL-8 in the culture supernatants were determined and compared to unstimulated cells (Basal) at six hours. Values are expressed as the mean ± SEM. * *P *< 0.05, significantly different from control as determined using Wilcoxon Signed Rank analysis.

To determine the effect of the TLR2 agonist Pam3CSK4 on induction of cytokine secretion in RA cells types, the expression of pro-inflammatory cytokines IL-6 and IL-8 were assessed. Figure 1A and 1B demonstrates in RA SFMC and PBMC, significant induction of IL-6 and IL-8 secretion following Pam3CSK4 (200 ng/ml) stimulation (*P *< 0.05). Incubation of PBMC and SFMC with Pam3CSK4 at 1 and 10 μg/ml also significantly induced IL-6 and IL-8 production (*P *< 0.05) (data not shown).

**Figure 2:**
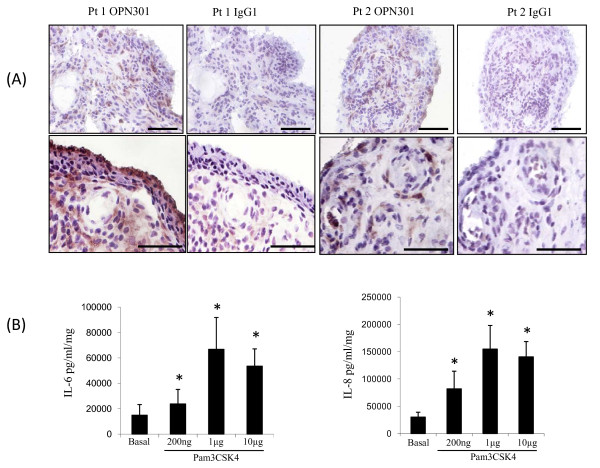
**TLR2 expression is localized to the perivascular region and lining layer**. **(A)** Two representative photomicrograph (Pt 1 and Pt 2) of RA synovial tissue of 10 patients stained for TLR2 expression. Expression is localized to the perivascular region and to the lining layer, with no staining observed for matched IgG control. The bar on the lower right hand corner of each photomicrograph represents a distance of 100 μm on the top panel and 50 μm on the bottom panel. **(B)** RA synovial tissue explant cultures were stimulated with TLR2 angonist Pam3CSK4 at 200 ng, 1 and 10 μg/ml. Levels of IL-6 (*n *= 11) and IL-8 (*n *= 8) in the culture supernatants were determined after 24 hrs. Values are expressed as the mean ± SEM. * *P *< 0.05 significantly different from control as determined using Wilcoxon Signed Rank analysis.

In RA synovial biopsies, TLR2 was localized to the lining layer and perivascular regions as demonstrated by staining using OPN301 as a primary antibody (Figure 2A). In paired RA synovial explant cultures, Pam3CSK4, at 200 ng/ml, 1 and 10 μg/ml significantly induced IL-6 and IL-8-cytokine production (Figure 2B). IL-6 production (mean pg/ml/mg ± SEM) increased from 14,934.9 ± 8,337.15 for an unstimulated control (Basal) to 23,783.02 ± 11,425.55 for Pam3CSK4 200 ng, *P *= 0.005; 66,811.71 ± 25,049.62 for Pam3CSK4 1 μg, *P *= 0.000 and 53,560.05 ± 13,547.85 for Pam3CSK4 10 μg, *P *= 0.000, respectively. IL-8 production (mean pg/ml/mg ± SEM) increased from 30,196.87 ± 8,871.838 for unstimulated control (Basal) to 81,931.94 ± 32,328.61 for Pam3CSK4 200 ng, *P *= 0.012; 154,736.9 ± 43,381.3 for Pam3CSK4 1 μg, *P *= 0.000 and 140,520.4 ± 28,061.14 for Pam3CSK4 10 μg, *P *= 0.004, respectively.

### Effect of OPN301 on cytokine production in PBMC and SFMC

**Figure 3:**
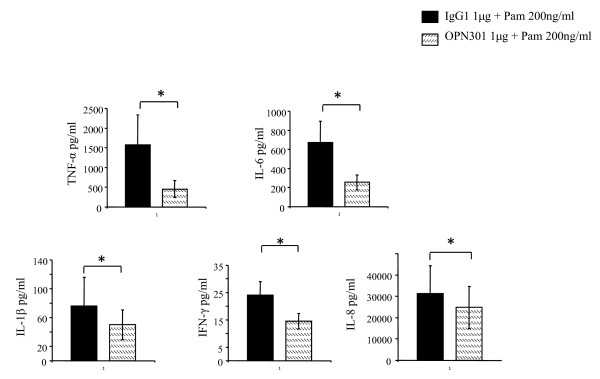
**OPN301 significantly inhibited cytokine production in SFMC compared to IgG isotype control**. RA SFMCs (*n *= 6) were stimulated with Pam3CSK4 in the presence or absence of OPN301 (1 μg/ml) or matched IgG isotype control for six hours. Values are expressed as the mean ± SEM. * *P *< 0.05, significantly different from control as determined using Wilcoxon Signed Rank analysis.

To assess whether OPN301 inhibits Pam3CSK induced cytokine secretion, RA PBMC and SFMC were stimulated with Pam3CSK4 (200 ng/ml) in the presence or absence of OPN301 or matched IgG isotype control. OPN301 significantly inhibited Pam3CSK4 induced IFN-γ, IL-1β, IL-6, TNF-α and IL-8 in RA SFMCs (all *P *< 0.05) compared to IgG isotype control (Figure 3). Similar results were obtained for RA PBMCs, where OPN301 significantly inhibited IFN-γ, IL-1β, TNF-α, IL-8, IL-6 cytokine production (all *P *< 0.05).

### OPN301 penetration and its effect on spontaneous cytokine production in RA synovial tissue explant cultures

**Figure 4:**
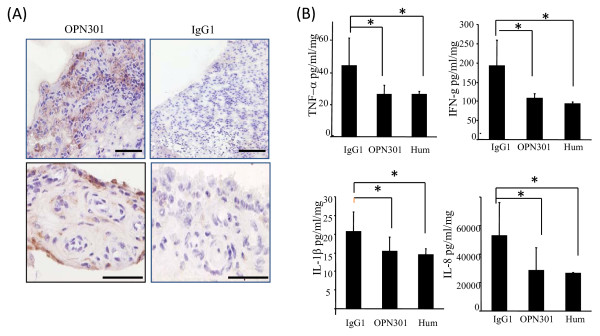
**OPN301 inhibition of spontaneous cytokine release is comparable to that observed for Adalimumab**. **(A)** One representative image of OPN301 penetration of RA synovial explant cultures of four RA patients. OPN301 was localized to the perivascular and lining layer regions with no staining observed for IgG control. The bar on the lower right hand corner of each photomicrograph represents a distance of 100 μm on the top panel and 50 μm on the bottom panel. **(B)** OPN301 (1 μg/ml) inhibited spontaneous cytokine release from RA synovial explant cultures (*n *= 13) and effect that was comparable to that observed anti-TNFα mAb Adalimumab (Hum, 1 μg/ml). Results are expressed as mean ± SEM. * P < 0.05 and ** P < 0.001 significantly different from control as determined using Wilcoxon Signed Rank analysis.

To show that OPN301 penetrates the RA ST in culture, biopsies were snap frozen following 72 h incubation with OPN301, sectioned and immunohistology performed omitting the primary antibody. Representative images of OPN301 detection in RA synovial explants are shown in Figure 4A. OPN301 was localized to the lining layer and to the perivascular regions, showing that OPN301 does penetrate the tissue in culture with localization consistent with previous studies [4-6].

To determine the inhibitory effect of OPN301 on spontaneous cytokine production by ST explant cultures, RA ST explants were established and cultured in the presence of OPN301 (1 μg/ml), Adalimumab (1 μg/ml) or IgG isotype control (1 μg/ml) for 72 hr (Figure 4B). OPN301 significantly inhibited spontaneous release of IL-8 (*P *= 0.001), TNF-α (*P *= 0.003), IFN-γ (*P *= 0.013), and IL-1β (*P *= 0.039). OPN301 inhibited IL-6 production, but this did not reach significance (*P *= 0.056). Adalimumab significantly inhibited IL-6 (*P *= 0.002), IL-8 (*P *= 0.000), IL-1β (*P *= 0.020), TNF-α (*P *= 0.018), and IFNγ (*P *= 0.001). The effect of OPN301 and Adalimumab on spontaneous cytokine production was also analysed using non-parametric Friedman analysis of variance, which further confirmed significant inhibition of all cytokines in response to both antibodies. We categorized the cytokine responses in the tissue cultures as follows, low response: <20% inhibition, moderate response: 21 to 49% inhibition, and good response: >50% inhibition. For OPN301, 38% of patients had a low response, 32% had a moderate response and 30% had a good response, while biopsies treated with Adalimumab showed 37% low response, 31% moderate response and 32% good response. The rate of response reflects what is observed for TNFα in clinical practice, (approximately 30% little or no response, and 70% of patient responses varying between 20 to 100%). Therefore, the results of our *ex vivo *model very much reflect typical biologic response rates for RA patients in clinical practice, and thus further validates the model as a good screening method for pre-clinical 'proof of concept studies'.

## Discussion

TLRs are phylogenetically conserved receptors involved in the innate immune response to microbial pathogens through recognition of pathogen associated molecular patterns (PAMPs). Recent studies have shown that TLRs recognize endogenous ligands, found in RA serum and synovial fluid [15,16], these ligands can be released from necrotic cells during tissue damage or cell stress, leading to TLR mediated immune responses [17-19]. Expression and activation of TLR 2, 3, 4 and 9 in RA ST has been demonstrated suggesting TLRs may be involved in the pathogenesis [4,20].

In this study we demonstrated that the TLR2 agonist Pam3CSK4 significantly upregulated pro-inflammatory cytokine production in RA mononuclear cells, an effect that was significantly blocked by OPN301. This is consistent with previous studies using RA FLS, where PG, a bacterial derived TLR2 agonist, significantly induced angiogenic factors, pro-inflammatory chemokines and cytokines [21-23]. This effect was inhibited by approximately 40% in the presence of an anti-TLR2 mAb [23]. Furthermore, studies using dominant negative forms of the essential TLR2/4 adapter molecules MyD88 and MAL/TIRAP also showed a partial inhibitory effect, which varied considerably depending on the cytokine analyzed, suggesting intricate signaling pathways are involved in this complex inflammatory environment [6]. A key role for TLR2 in RA is further supported by evidence from animal models. TLR2 and MyD88 knockout mice are protected from SWC induced joint inflammation [24]. Intra-articular injection of the TLR2 and NOD2 ligand PG led to development of destructive arthritis in mice [8]. Functional studies have shown that stimulating TLR2 expressing RA fibroblasts with PG, leads to induction of cytokines and matrix-metalloproteinases [23]. TLR2 has also been implicated in other inflammatory diseases such as atherosclerosis and inflammatory bowel disease [25,26].

TLR2 expression was localized to synovial tissue lining and sub-lining layers of RA patients, which is consistent with previous studies. *In situ *hybridization revealed TLR2 mRNA expression in the synovial lining, small vessels and in areas of infiltrating lymphocytes [4]. TLR2 and TLR4 expression has also been shown in the lining, sublining and perivascular regions of RA synovial tissue, with TLR2 expression higher than that of TLR4 [23]. TLR2 is also expressed at the sites of attachment and invasion into cartilage and bone [4]. Furthermore, in this study we show that OPN301 directly penetrated RA synovial explant cultures, localizing to the lining layer, which is comprised of synoviocyte-like fibroblasts and macrophages and to the perivascular region where angiogenesis and leukocyte extravasation occurs, critical mechanisms in the pathogenesis of RA. Consistent with this localization, TLR2 is functionally active in synovial fibroblasts and endothelial cells, where its activation results in induction of VEGF, IL-8, ICAM-1, VCAM-1 and MMPs [15,21,22,27-29]. Moreover, several studies have demonstrated that TLR2 activation of monocytes resulted in an increase in adhesive and migratory capacity of cells [29].

In this study we used an *ex vivo *RA ST explant model to investigate the role of TLR2 blockade in RA synovial inflammation. This model more closely reflects the *in vivo *joint environment, as it maintains tissue architecture and cell-cell contact of the complex mix of different cell types whose interaction contributes to the pro-inflammatory environment in the RA joint. Furthermore, RA synovial explants spontaneously release key pro-inflammatory cytokines and, therefore, this model is ideal for examining potential therapeutic molecules. We demonstrated that OPN301 significantly inhibited spontaneous secretion of TNFα, IL-1β, IFNγ and IL-8, suggesting that TLR2 is important in RA pathogenesis. The inhibitory properties of OPN301 and Adalimumab on spontaneous release of proinflammatory cytokines in our explant model reflect response rates observed in routine clinical practice.

Activation of TLRs by local endogenous ligands leading to increased proinflammatory cytokine/chemokine secretion, may result in a vicious cycle of inflammation, ultimately leading to the pathological destruction of cartilage and bone seen in RA [2]. While bacterial TLR ligands have been found in RA synovial fluid and tissue, they have also been found in normal tissue [30]. Endogenous TLR ligands, which are released under inflammatory conditions and in response to tissue damage, have now been implicated in RA. Evidence for TLR4 ligands have been demonstrated, where RA synovial fluid stimulated TLR4 expressing CHO cells to regulate CD25 [31], and RNA released from necrotic synovial fluid can activate RA synovial fibroblasts in a TLR3 mediated mechansim [15]. While no ligand has been defined, the existence of a ligand is supported here and by other studies, which show that conditioned media from RA synovial explants can activate macrophages in a MyD88 and Mal dependent manner [6]. Several potential ligands have been suggested, such as Heat Shock Proteins, Fibronectin fragments, Hyaluronan oligosaccharides, HMGB1 and GP96; all of which have been identified in the RA joint [19,32-36].

## Conclusions

Targeting of the inflammatory cytokine TNF-α by biologic agents, such as Adalimumab, has been the most beneficial treatment strategy to date for patients with arthritis [9]. However, a significant proportion of patients fail to respond to these therapies, while others may be at risk of adverse events such as infections due to impaired immune function [9]. Our findings support further evaluation of strategies targeting TLR2 as potential therapeutic agents for the treatment of RA.

## Abbreviations

CRP: C-reactive protein; DAS28: 28-joint count Disease Activity Score; DMARDs: disease-modifying antirheumatic drugs; EBAO: ethidium bromide acridine orange solution; ECM: extracellular matrix; ESR: erthrocyte sedimentation rate; FLS: synovial fibroblasts; mAb: monoclonal antibody; NSAIDs: nonsteroidal anti-inflammatory drugs; PAMPs: pathogen associate molecular patterns; PBMC: peripheral blood mononuclear cells; PG: petidoglycan; RA: rheumatoid arthritis; SFMC: synovial fluid mononuclear cells; ST: synovial tissue; SVUH: St. Vincent's University Hospital; TLR: Toll-like receptor; VAS: visual analog scale.

## Competing interests

DV is in receipt of a research grant from Opsona Therapeutics Ltd. Current Opsona employees (BK, PMcG, MR, WMcC) and ex-Opsona employee JD hold shares in Opsona Therapeutics Ltd; however, the percent involved is so small that Opsona does not view this as a conflicting interest. Luke O'Neill is a founder of Opsona Therapeutics Ltd. PMcG and BK are named inventors on patent WO2009/000929 and Jerome Dellacasagrande is named inventor on patent PCT/EP2010/059667. SNAU, TS, JMcC, MC and UF have no competing interests.

## Authors' contributions

SNAU conducted most of the experiments and analysis of data. TS, JMcC, MC and UF performed some of the experiments. UF, DV, MC, PMcG, SNAU, JD, BK, WMcC, MR, TS and LO'N, participated in the study design, data analysis and manuscript preparation. UF and DV supervised the research. DV and TS recruited all patients, performed the arthroscopies and provided all clinical information. All authors read and approved the final manuscript.
